# S100A16 promotes differentiation and contributes to a less aggressive tumor phenotype in oral squamous cell carcinoma

**DOI:** 10.1186/s12885-015-1622-1

**Published:** 2015-09-09

**Authors:** Dipak Sapkota, Ove Bruland, Himalaya Parajuli, Tarig A. Osman, Muy-Teck Teh, Anne C. Johannessen, Daniela Elena Costea

**Affiliations:** 1 Department of Clinical Medicine, The Gade Laboratory for Pathology, University of Bergen, Haukeland University Hospital, N-5021 Bergen, Norway; 2 Centre for Cancer Biomarkers (CCBIO), Faculty of Medicine and Dentistry, University of Bergen, N-5021 Bergen, Norway; 3 Center of Medical Genetics and Molecular Medicine, Haukeland University Hospital, University of Bergen, N-5021 Bergen, Norway; 4 Centre for Clinical and Diagnostic Oral Sciences, Institute of Dentistry, Barts and The London School of Medicine and Dentistry, Queen Mary University of London, England, UK; 5 Department of Pathology, Haukeland University Hospital, Bergen, Norway

## Abstract

**Background:**

Altered expression of S100A16 has been reported in human cancers, but its biological role in tumorigenesis is not fully understood. This study aimed to investigate the clinical significance and functional role of S100A16 in oral squamous cell carcinoma (OSCC) suppression.

**Methods:**

*S100A16* mRNA and/or protein levels were examined by quantitative RT-PCR and immunohistochemistry in whole- and laser microdissected-specimens of normal human oral mucosa (NHOM, *n* = 65), oral dysplastic lesions (ODL, *n* = 21), OSCCs (*n* = 132) and positive cervical nodes (*n* = 17). S100A16 protein expression in OSCC was examined for correlations with clinicopathological variables and patient survival. S100A16 was over-expressed and knocked-down in OSCC-derived (CaLH3 and H357) cells by employing retroviral constructs to investigate its effects on cell proliferation, sphere formation and three dimensional (3D)-organotypic invasive abilities *in vitro* and tumorigenesis in a mouse xenograft model.

**Results:**

Both *S100A16* mRNA and protein levels were found to be progressively down-regulated from NHOM to ODL and OSCC. Low S100A16 protein levels in OSCC significantly correlated with reduced 10-year overall survival and poor tumor differentiation. Analysis of two external OSCC microarray datasets showed a positive correlation between the mRNA expression levels of *S100A16* and keratinocyte differentiation markers. CaLH3 and H357 cell fractions enriched for differentiated cells either by lack of adherence to collagen IV or FACS sorting for low p75NTR expression expressed significantly higher *S100A16* mRNA levels than the subpopulations enriched for less differentiated cells. Corroborating these findings, retroviral mediated S100A16 over-expression and knock-down in CaLH3 and H357 cells led to respective up- and down-regulation of differentiation markers. *In vitro* functional studies showed significant reduction in cell proliferation, sphere formation and 3D-invasive abilities of CaLH3 and H357 cells upon S100A16 over-expression. These functional effects were associated with concomitant down-regulation of self-renewal (Bmi-1 and Oct 4A) and invasion related (*MMP1* and *MMP9*) molecules. S100A16 over-expression also suppressed tumorigenesis of H357 cells in a mouse xenograft model and the resulting tumor xenografts displayed features/expression of increased differentiation and reduced proliferation/self-renewal.

**Conclusions:**

These results indicate that S100A16 is a differentiation promoting protein and might function as a tumor suppressor in OSCC.

**Electronic supplementary material:**

The online version of this article (doi:10.1186/s12885-015-1622-1) contains supplementary material, which is available to authorized users.

## Background

Oral squamous cell carcinoma (OSCC) is an aggressive neoplasm which is highly invasive and frequently metastasizes to cervical lymph nodes leading to a severely reduced patient survival. Despite recent advances in diagnosis and treatment modalities, less than 50 % of OSCC patients survive for 5 years [1]. Among the molecular and cellular changes occurring during OSCC development, a significant disturbance in cellular differentiation and maturation process has been reported to be a common event in oral carcinogenesis [2–4]. Nevertheless, the precise molecular mechanism regulating differentiation and its contribution to OSCC progression is not fully understood.

The S100 protein family is a multifunctional group of EF-hand calcium binding proteins. This family consists of small acidic proteins (10–12 kDa) that are expressed only in vertebrates in a cell and tissue specific manner. To date, 25 S100 protein members have been described in humans [5, 6]. Genes encoding several of the members of this family are clustered in the epidermal differentiation complex (EDC) on chromosome 1q21 [7–9], and many of the S100 members have been reported to be involved in cellular differentiation and differentiation-related pathologies [10, 11]. In addition, S100 proteins have recently been implicated in the regulation of epithelial-mesenchymal transition, cancer stem cells and tumor heterogeneity in human malignancies [12–14].

S100A16 is a recent addition to the S100 protein family [15]. Although it has been reported to be widely expressed in human tissues [15], its precise biological functions are not fully understood. In a recent study, S100A16 has been suggested to be related with cell invasion and poor prognosis in human breast cancer [16]. We have identified S100A16 to be an interaction partner of S100A14, a proliferation and invasion-related protein in OSCC [17–19]. These observations indicate that S100A16 might be related with OSCC progression. Nevertheless, functional roles and prognostic significance of this protein are currently unknown in OSCC. In the current study, we demonstrate that down-regulation of S100A16 expression in OSCC specimens was associated with poor prognosis and poor differentiation grade. Experimentally, S100A16 was found to promote malignant keratinocyte differentiation and to suppress aggressive tumor phenotype such as proliferation, sphere formation and 3D-organotypic invasive abilities of OSCC-derived cells *in vitro* and tumorigenesis in a mouse xenograft model.

## Methods

### Human tissue specimens

All tissue samples were collected from Haukeland University Hospital after informed written patient consent. This study was approved by the Committee for Medical and Health Research Ethics in West Norway (2011/1244 REK vest, 2010/481 REK vest). A total number of 75 normal human oral mucosa [NHOM, 31 formalin fixed-paraffin embedded (FFPE) and 44 frozen], 21 oral dysplastic lesion (ODL, all FFPE), 132 OSCC (82 FFPE and 50 frozen) and 17 positive cervical lymph nodes (all FFPE) were used in the current study for the expression analysis of S100A16 by immunohistochemistry (IHC) and/or quantitative RT-PCR (qRT-PCR). All OSCC patients included in the study were newly diagnosed cases, and had no history of chemo- or radiotherapy prior to surgery. All NHOM specimens were donated by patients undertaking wisdom tooth extraction. For S100A16 IHC, FFPE specimens of NHOM (*n* = 21), ODL (*n* = 11; 1 carcinoma *in situ*, 1 severe, 7 moderate and 2 mild dysplastic lesions), OSCCs (*n* = 65), and positive cervical lymph nodes (*n* = 17) were used. Details of the clinicopathological information of these OSCC cases are reported in Table 1. FFPE specimens of NHOM (*n* = 10), ODL (*n* = 10) and OSCC (*n* = 17) were laser microdissected and used for quantification of *S100A16* mRNA by qRT-PCR. In OSCC specimens, paratumor (dysplastic) epithelium, tumor center/core and the corresponding invading front/island were microdissected. Detailed methodology for laser microdissection is reported in Additional file 1. *S100A16* mRNA expression was examined in frozen tissues of normal human oral mucosa (NHOM, *n* = 44) and OSCCs (*n* = 50). These tissues were stored at −80 °C till RNA extraction.

**Table 1:** S100A16 expression (PLI score) and clinicopathological variables of the OSCC patients

	PLI score at invading fronts/islands^a^		
Variables	Low, *n* (%)	High, *n* (%)	*P*
Age^b^ (years)			
≤64	18 (60.0)	12 (40.0)	0.108
>64	14 (60.0)	21 (60.0)	
Gender			
Female	10 (47.6)	11 (52.4)	0.857
Male	22 (50.0)	22 (50.0)	
Location			
Tongue	14 (45.2)	17 (54.8)	0.566
Gingiva, buccal mucosa & oral lip	11 (47.8)	12 (52.2)	
Floor of mouth & oro-pharynx	7 (49.2)	4 (36.4)	
Differentiation			
Poor and moderate	22 (62.9)	13 (37.1)	0.018
Well	10 (33.3)	20 (66.7)	
Lymph node involvement			
Negative (N0)	15 (39.5)	23 (60.5)	0.062
Positive (N1 & N2)	17 (63.0)	10 (37.0)	
Tumor size			
T1 & T2	19 (52.8)	17 (47.2)	0.638
T3 & T4	13 (44.8)	16 (55.2)	
Recurrence			
No	20 (43.5)	26 (56.5)	0.149
Yes	12 (63.2)	7 (36.8)	
Tumor stage			
Early (1 & 2)	8 (38.1)	13 (61.9)	0.215
Late (3 & 4)	24 (54.5)	20 (45.5)	

^a^OSCCs were stratified into high and low S100A16 expression groups by using median S100A16 PLI score as a cut-off

^b^patients were categorized into low- and high-age groups based on the median age

### External microarray databases

Eight external microarray datasets, four for OSCC and head and neck SCC (mainly consisting of OSCC) [20–23], and one each for esophageal squamous cell carcinoma (ESCC) [24], colorectal carcinoma (CRC) [25], prostate cancer [26] and ovarian cancer [27] were used either i) to validate the down-regulation of *S100A16* in OSCC or in the above mentioned malignancies or ii) for the correlation analyses of *S100A16* and differentiation related molecules.

### IHC

S100A16 IHC was performed in FFPE tissue specimens of NHOM, ODL, OSCCs, and positive cervical lymph nodes as described previously [19]. Briefly, antigen retrieval was done by microwave treatment in Tris-EDTA buffer, pH 9.0 (DAKO). After blocking with 10 % goat serum, rabbit polyclonal anti-human S100A16 primary antibody (11456-1-AP, Proteintech, Chicago, IL, USA, 1:100 dilutions) was applied. After wash, anti-rabbit secondary antibody conjugated with horseradish peroxidase labeled polymer (EnVision System, DAKO) was applied. Presence of antigen was visualized by staining with 3, 3′-diaminobenzidine (DAKO), counterstained with hematoxylin (DAKO) and mounted with EuKit mounting medium. Sections incubated with 3 % BSA instead of primary antibody served as negative controls. FFPE tissues from mouse tumor xenografts were stained with anti-S100A16, anti-involucrin, anti-Ki67, and anti-Bmi-1. For detailed methodology of IHC and the antibody used, see Additional file 1.

### IHC evaluation

Blinded for the clinical information, IHC evaluation of all specimens was done at 400× (40× objective lens) using Leica DMLB microscope (Leica Microsystems). Inter-observer variation was controlled by calibrating the evaluation done by three investigators (DS, TAO and HP). Afterwards, all specimens were evaluated by one investigator (DS). Expression pattern of S100A16 was evaluated semiquantitatively by scoring three consecutive fields (>500 cells/field, whenever possible) on the surface epithelium of NHOM and ODL, and at the invading tumor islands of lymph nodes. For OSCCs, the evaluation was done both at the central and the invading front (the deepest part of an invasive tumor, >3–4 cell layers thick). When it was not possible to identify clear invasive fronts, deepest invading tumor islands consisting of >50 cells were used for quantification. A composite scoring system combining the number of S100A16 positive cells (P score), cellular localization (membranous or cytoplasmic or both, L score) and intensity (I score) was used for S100A16 scoring. The final (PLI) score was calculated by multiplying the individual P, L and I scores and averaging PLI scores of the three evaluated fields. For details of the PLI scoring system, see Additional file 1.

The evaluation of Ki67 staining in the tumor xenografts was done only at the invading fronts (5–6 cell layers). Positive and negative tumor cell nuclei were manually counted (at least 300 cells were counted in 3–6 representative areas, at 40× objective lens) and the fraction of the positive cells were calculated. Bmi-1, S100A16 and involucrin staining in the tumor xenografts were evaluated qualitatively only.

### Cell culture, construction of expression vector and transfection

The oral squamous cell carcinoma-derived cell-lines CaLH3 [28] and H357 [29] were cultured as described elsewhere [17]. S100A16 expression and shRNA vectors were constructed as described previously [17, 19]. For details of the expression and shRNA vector construction, see Additional file 1. CaLH3 and H357 cells infected with retrovirus with S100A16 insert and retrovirus without S100A16 insert are referred to as ‘S100A16-CaLH3 and S100A16-H357’, and ‘control-CaLH3 and control-H357’ cells, respectively.

### Tissue engineering (3D-models) and evaluation of carcinoma cell invasion

Primary carcinoma associated fibroblasts isolated from a patient with OSCC were embedded in collagen type I biomatrix (BD Biosciences), and seeded on top with control or S100A16 over-expressing CaLH3 cells, as previously described [30]. 3D constructs were harvested, formalin-fixed and paraffin-embedded. Depth of invasion was measured on 5-μm sections stained with hematoxylin and eosin using Olympus DP.Soft 5.0 software. For the measurement of carcinoma cell invasion, each 3D-organotypic section was divided into fifths. The central and the two outer fifths were excluded from measurements, depth of invasion being assessed in the remaining two fifths only. For this, a horizontal line was drawn (using the software Olympus DP.Soft 5.0) through the uppermost remnants of the collagen gel to visualize the basement membrane zone; depth of invasion was determined every 100 μm along this horizontal line as the vertical distance from this line to the limit of invading epithelial cells (Fig. 5f).

### RNA extraction, cDNA synthesis and qRT-PCR

RNA was extracted from frozen specimens (NHOM and OSCC), laser microdissected FFPE tissues (NHOM, ODL and OSCC) and OSCC-derived cell-lines respectively using Dynabeads mRNA Direct kit (Invitrogen), RNeasy FFPE Kit (#73504, Qiagen) and RNeasy fibrous tissue mini kit (cat no: 74704, Qiagen Inc.). See Additional file 1: Supplementary methods and Table S2 for details of the cDNA synthesis and qRT-PCR.

### Immunoblotting

Twenty to 30 μg of cell lysates were resolved in NuPAGE® Novex 4–12 % Bis-TrisTris gel (NP0329, Life technologies, NY, USA) and immunoblotted with antibodies as described in Additional file 1: Table S3.

### Real time cell proliferation assay (xCELLigence system)

The xCELLigence DP device from Roche Diagnostics (Mannheim, Germany) was used to quantitatively and dynamically monitor cell proliferation in real-time. Six thousands control or S100A16 over-expressing CaLH3 and H357 cells were seeded in duplicates in the electronic microtiter E-plates (Cat. No: 5469830001; Roche Diagnostic) and proliferation was measured in real time for 72 h. Data acquisition and analysis was performed with the RTCA software (version 1.2.1.1002, Roche Diagnostics).

### *In vitro* sphere formation assay

Inner surface of each well of 48 well-plate was coated evenly with a 12 mg/mL solution of polyHEMA (sigma, P3932) in 95 % ethyl alcohol and sterilized under UV overnight. Afterwards, 490 μL of cell culture medium with 1 mg/mL methylcellulose was added in each well. One thousand cells suspended in 10 μL medium was then added in each well and evenly mixed with the medium. Sphere formation was quantified on 14th day by counting the number of spheres (>50 cells) at 4× objective under Nikon ECLIPSE TS100 fluorescent microscope. Each experiment was repeated thrice in 6 replicates.

### Adherence to collagen IV

Previous studies have shown that rapid adherence of keratinocytes to collagen IV is a robust method to enrich cells for stem cell properties [31, 32]. According to this method, cells adhering most rapidly to collagen IV are considered to be enriched for cells with a less differentiated phenotype (stem cell properties); whereas the late adherent cell population contains relatively fewer cells with stem cell properties and the non-adherent cell population consists of cells with a more differentiated phenotype. This assay was performed as described previously [33, 34]. Briefly, cell suspension was allowed to attach to culture dishes coated with collagen IV (10 μg/mL) (BD Biosciences, USA) in the cell incubator for 10 min. Cells attached to the dishes were collected and referred to as rapid adherent cells (RAC). The unattached cells within the first 10 min were then transferred to a new collagen IV-coated dish for an additional 30 min in incubator. Cells that adhered within this period were referred to as middle adherent cells (MAC). Remaining unattached cells were collected as late adherent cells (LAC).

### Fluorescent activated cell sorting (FACS) for p75NTR and cytokeratin 13

p75NTR, a member of tumor necrosis factor receptor superfamily, is a low affinity neurotrophin receptor. Accumulated evidences suggest that p75NTR is a putative stem cell marker both in the normal oral and esophageal tissues [35–37] as well as in the malignancies including OSCC [37–40]. Accordingly, cells with p75NTR high expression are considered to be enriched for cells with a less differentiated phenotype (stem cell properties), whereas the cells with low P75NTR expression are enriched for cells with a more differentiated phenotype. Unfixed oral cancer cells were stained with anti-p75NTR antibody (Sigma Aldrich, 1:250 dilutions) whereas methanol fixed cells were stained with anti-cytokeratin 13 antibody (Novacastra, 1:350 dilutions). For detailed methodology of FACS, see Additional file 1.

### *In vivo* tumorigenesis assay

Protocols for all animal studies were approved by the Norwegian Animal Research Authority (Project ID: 20124236). Twelve nonobese diabetic/severe combined immunodeficient (NOD/SCID) mice were randomly divided into two groups (*n* = 6, each group). One thousand S100A16-H357 or control-H357 cells suspended in 50 μL of Matrigel (BD Biosciences) were injected in the tongue of each mouse. Tumor development was monitored regularly under inhalation anesthesia. Length and breadth of the formed tumors were measured by Vernier caliper and tumor volume was calculated using the following formula-(length × breadth^2^)/2. Tumor formation was confirmed histologically.

### Statistics

Statistical analysis was done using SPSS 21 and/or GraphPad prism 5. Difference in means between two groups was analyzed by using unpaired *t*-tests, whereas comparison between more than two groups was done by using ANOVA test with Bonferroni Post-Hoc. Median PLI scores both at the tumor center and at the invading front/island were used as cut-off values to stratify OSCCs into high- and low-S100A16 expression groups. According to the differentiation status, OSCCs were categorized into two groups: highly differentiated and moderately-poorly differentiated. Association between the expression status of S100A16 and other binary variables was done using Chi-square Test. Survival analysis was performed using the Kaplan-Meier analysis (log-rank test). Cox proportional hazard model was used to examine the effect of S100A16 expression on 10-year overall survival. Level of significance was set at 5 %.

## Results

### S100A16 was progressively down-regulated from normal tissue to dysplasia and OSCC; and low S100A16 expression at the invading front/islands correlated with reduced survival and poor tumor differentiation

**Fig. 1:**
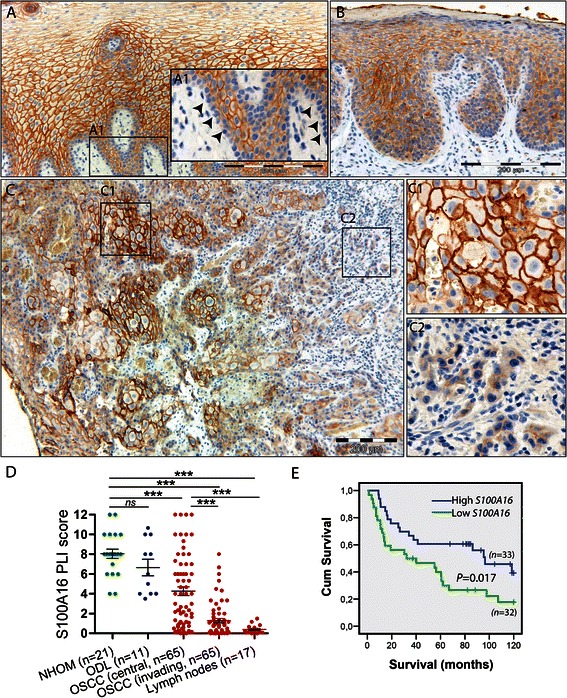
S100A16 protein was progressively down-regulated from NHOM to ODL and OSCC and low S100A16 protein expression correlated with poor OSCC prognosis. **a** Representative NHOM specimen showed strong, predominantly membranous S100A16 expression in the epithelial compartment. Basal cell layer (arrowheads), however, was mostly negative for S100A16 expression (*A1*). **b** Expression pattern of S100A16 in ODL was similar to that of NHOM. However, the expression intensity was weaker than that in NHOM (**c**) Representative OSCC lesion showing a gradient of S100A16 expression: central area (*C1*) showed a strong, membranous staining in contrast to a very weak, mostly cytoplasmic staining in the invading front area (*C2*). **d** Graphic illustration of S100A16 PLI score demonstrated gradual down-regulation of S100A16 from NHOM to ODL, OSCC and positive cervical nodes. ANOVA test with Bonferroni Post-Hoc was used for the statistical analysis. *P*-value: ***, <0.001; ns, not significant. **e** Kaplan-Meier curves showing reduced 10-year survival probabilities for patients with low S100A16 PLI score. Log-Rank test was used for statistical analysis

To examine the expression and localization of S100A16, IHC was performed on archived FFPE specimens of NHOM (*n* = 21), ODL (*n* = 11), OSCC (*n* = 65) and positive cervical lymph nodes (*n* = 17). A strong membranous expression of S100A16 was found in the supra-basal (committed/differentiating) epithelial cell layers of all NHOM tissues (Fig. 1a). Negative or weak cytoplasmic staining was found in the basal cell layer (stem cell compartment) in most of NHOM samples (Fig. 1a and A1). The expression pattern of S100A16 in ODL was similar to that found in NHOM (Fig. 1b). The superficial and central areas of OSCC specimens demonstrated similar staining pattern to that found in NHOM, whereas very weak or negative expression was observed at the invading front/island of tumor cells with concomitant membrane to cytoplasmic translocation in majority of the cases (Fig. 1 C1 and C2). Nevertheless, S100A16 staining was relatively strong with membranous localization at the invading front/island of well-differentiated OSCCs (Additional file 2: Figure S1A). S100A16 staining was very weak or absent in the infiltrating tumor islands of positive cervical lymph nodes (Additional file 2: Figure S1B).

Quantification of S100A16 staining showed that S100A16 PLI score was gradually decreased during the transition from NHOM to ODL and OSCC (Fig. 1d). Of note, PLI score was found to be lower at the invading front/island as compared to the central areas in OSCCs (Fig. 1d). Examination of possible correlation between S100A16 expression and clinical parameters showed that low S100A16 PLI score at the invading front/island was associated with reduced 10-year overall survival (Log-Rank test, *P* = 0.017) (Fig. 1e), moderate-poorly differentiated OSCCs (*P* = 0.018) and lymph node involvement (*P* = 0.062) (Table 1). Multivariate Cox regression analysis demonstrated that S100A16 expression was a significant prognostic factor (HR = 0.483, CI = 0.24–0.95, *P* = 0.037) for the survival of OSCC patients (Table 2). However, no significant correlations were observed between the PLI score at the tumor center and clinicopathological variables (Additional file 1: Table S1). A trend for better survival probabilities was found for well differentiated and early stage tumors, but the results were not statistically significant (data not shown).

**Table 2:** Results of a multivariate Cox regression analysis for predicting the overall survival of OSCC cases

Variables	Assigned score	Hazard ratio	95 % CI	*P*-value
Age				
≤64	0	1.51	0.82–2.94	0.169
>64	1			
Sex				
Female	0	1.18	0.60–2,33	0.623
Male	1			
Differentiation				
Well	0	1.09	0.53–2.94	0.803
Moderate & poor	1			
T-stage				
T1 & T2	0	0.871	0.39–1.90	0.728
T3 & T4	1			
Clinical stage				
Early (1 & 2)	0	1.371	0.55–3.33	0.494
Late (3 & 4)	1			
S100A16				
High	0	0.483	0.24–0.95	*0.037*
Low	1			

*CI*, Confidence interval

### *S100A16* mRNA level was progressively down-regulated from NHOM to ODL and OSCC

**Fig. 2:**
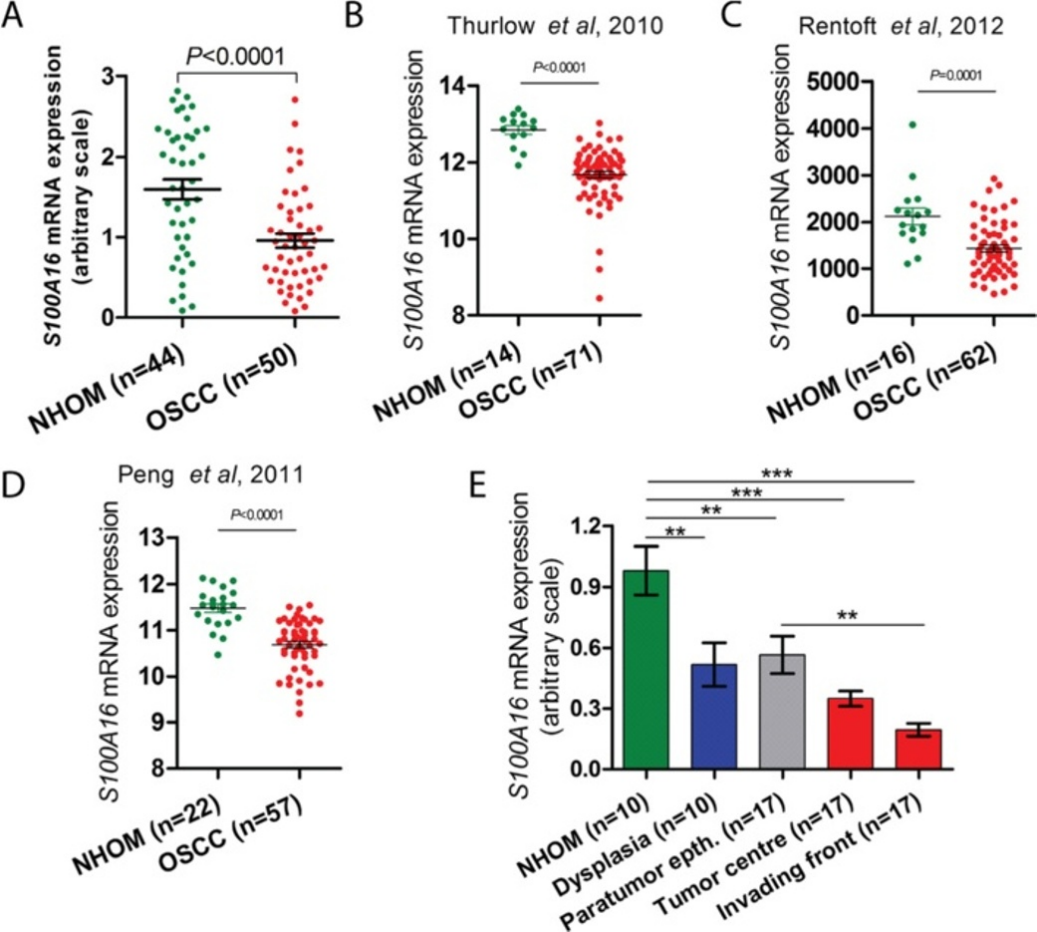
*S100A16* mRNA level was progressively down-regulated from NHOM to ODL and OSCC. **a** *S100A16* mRNA expression was examined in frozen specimens of NHOM (*n* = 44) and OSCCs (*n* = 50) by using qRT-PCR. Mean *S100A16* mRNA was found to be significantly down-regulated in OSCCs (*P* < 0.0001). *S100A16* mRNA expression levels were normalized to *GAPDH* mRNA expression. Error bars represent SEM. Student’s-*t* test was performed for statistical analysis. **b**–**d** Down-regulation of *S100A16* mRNA levels in OSCC was verified in three independent microarray datasets. Error bars represent SEM. Student’s-*t* test was performed for statistical analysis. **e** Gradual down-regulation of *S100A16* mRNA during the transition from NHOM to ODL and OSCC was validated in laser dissected specimens of NHOM, ODL, paratumor (dysplastic) epithelium, tumor center and invading front by qRT-PCR. qRT-PCR was done in duplicates and *S100A16* mRNA level was normalized to *GAPDH* and *ACTB* mRNA levels. Error bars represent SEM. ANOVA test with Bonferroni Post-Hoc was used for the statistical analysis. *P*-value: ***, <0.001; **, 0.001–0.01

Expression levels of *S100A16* mRNA were quantitatively examined in an independent cohort of frozen specimens of NHOM (*n* = 44) and OSCC (*n* = 50) by qRT-PCR. The mean expression of *S100A16* mRNA was found to be significantly down-regulated in OSCC compared to NHOM (*P* < 0.0001) (Fig. 2a). Down-regulation of *S100A16* mRNA levels was verified in three independent microarray datasets for OSCC (Fig. 2b–d). To validate the progressive down-regulation of *S100A16* mRNA expression during OSCC progression, FFPE specimens of NHOM, ODL and OSCC were laser dissected and mRNA levels were quantitatively examined. Parallel to the IHC findings, mRNA expression level was progressively down-regulated in the oral keratinocytes during the transition from NHOM to ODL, including paratumor epithelium, and OSCC (Fig. 2e).

### *S100A16* mRNA level was down-regulated during tumor progression of several other human malignancies

To investigate whether S100A16 down-regulation is a common event during tumor progression of other carcinomas as well, the expression levels of *S100A16* mRNA were examined in external microarray datasets of other human malignancies and tumor progression model systems. Similar to OSCC, *S100A16* mRNA level was found to be significantly down-regulated in ESCC and CRC as compared to the corresponding control specimens (Additional file 3: Figure S2A-B). Moreover, progressive down-regulation was observed during various stages of tumor progression in prostate cancer and in ovarian cancer model systems (Additional file 3: Figure S2C-D).

### *S100A16* mRNA expression was positively correlated with differentiation markers in OSCC specimens and in cell fractions enriched for differentiated cells

**Fig. 3:**
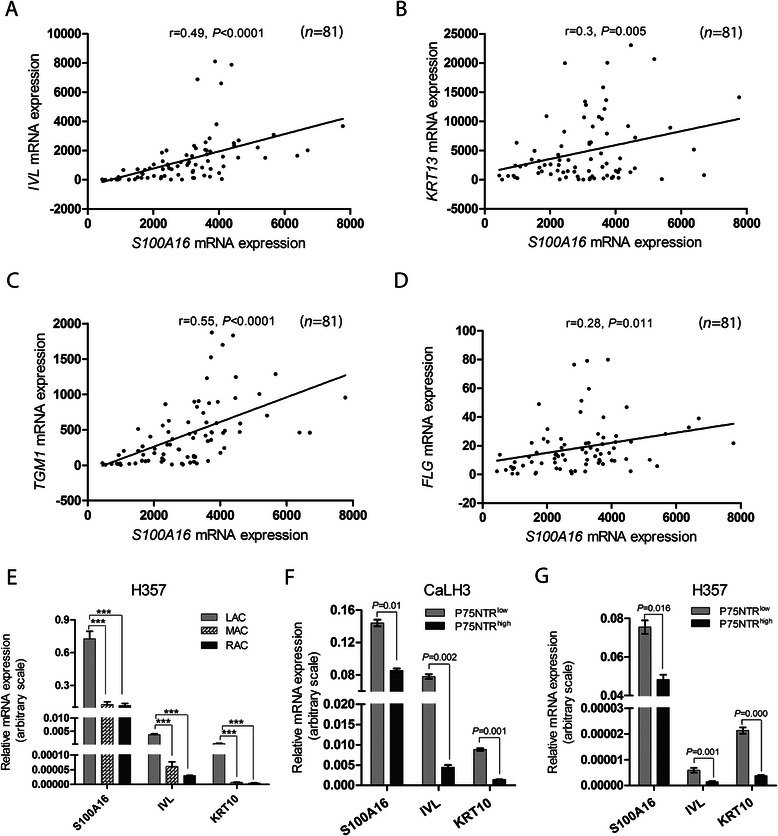
*S100A16* mRNA expression was positively correlated with differentiation markers in OSCC specimens and in the cell fractions enriched for more differentiated cells. **a–d** *S100A16*, *IVL*, *KRT13*, *TGM1* and *FLG* mRNA levels were obtained from external microarray dataset (Rickmen) and their correlation was examined using Pearson analysis. **e–g** Cell fractions were enriched for differentiated cells either by using lack of adherence to collagen IV or by FACS sorting for low p75NTR expression and mRNA expression levels of *S100A16* and *IVL* and *KRT10* were examined by qRT-PCR. **e** Significantly higher mRNA levels of *S100A16*, *IVL* and *KRT10* were found in LAC cell fractions (enriched for more differentiated cells) as compared to RAC/MAC (enriched for less differentiated cells). Error bars represent SEM of 3 repeated experiments. ANOVA test with Bonferroni Post-Hoc was used for statistical analysis. *P*-value: ***, <0.001. **f** and **g** Fractions enriched for differentiated cell (p75NTR^low^) expressed significantly higher expression of *S100A16*, *IVL* and *KRT10* as compared to p75NTR^high^ fractions in CaLH3 (**f**) and H357 (**g**) cells. Expression levels were normalized to *GAPDH* mRNA expression. Error bars represent SEM of 3 repeated experiments. Student’s-*t* test was performed for statistical analysis

Positive correlation between the expression of S100A16 as examined by IHC and the differentiation status found in the OSCC specimens prompted us to further examine the correlation between *S100A16* and differentiation markers in OSCC specimens *in vivo*, and in the differentiated cell fractions *in vitro. S100A16* mRNA levels were positively correlated with mRNA levels of several of the differentiation markers (*IVL*, *KRT13*, *TGM1* and *FLG*) in two independent microarray datasets [20, 21] (Fig. 3a–d and Additional file 4: Figure S3). In parallel, similar correlation was also found in the LAC and p75NTR^low^ cell fractions (enriched for differentiated cells) compared to the RAC/MAC and p75NTR^high^ fractions (enriched for less differentiated cells) (Fig. 3e–g).

### S100A16 modulated expression of differentiation-related markers in OSCC-derived cells

**Fig. 4:**
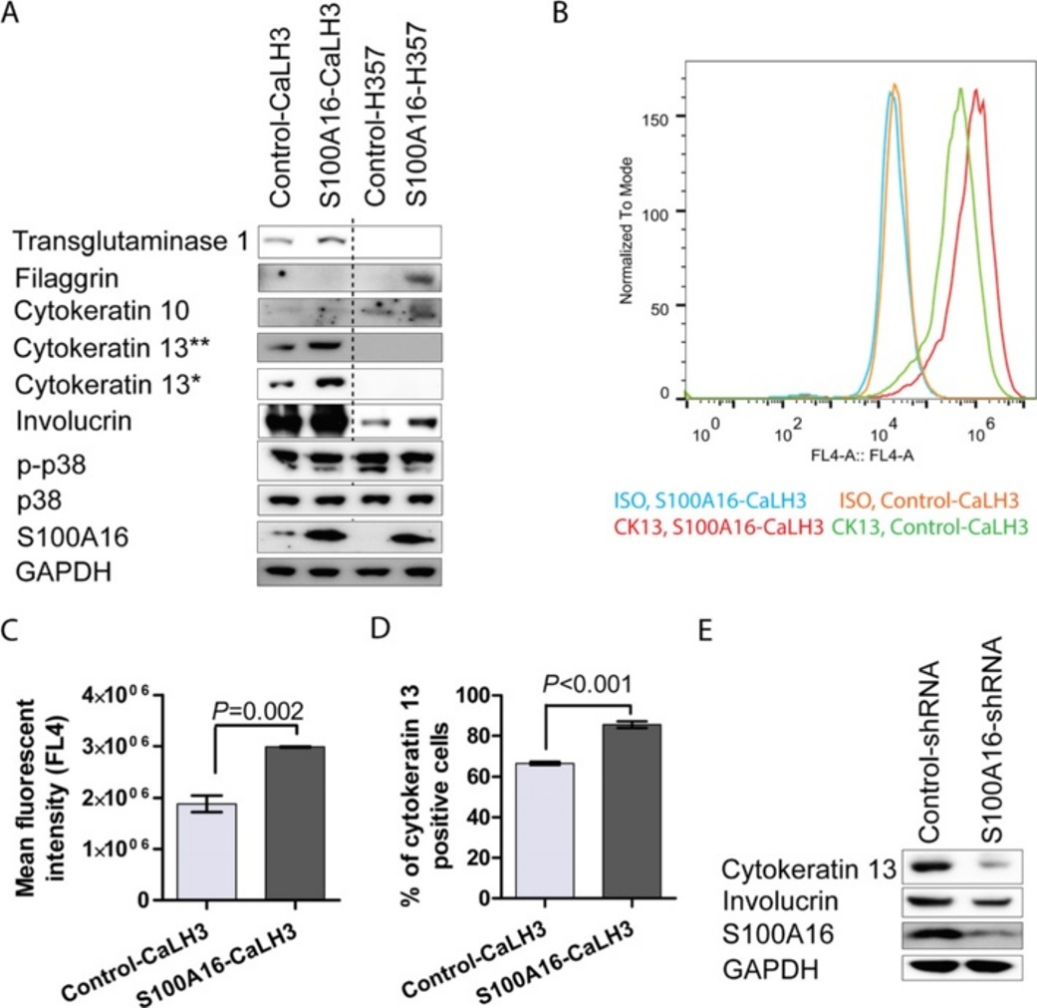
S100A16 over-expression modulated differentiation-related markers in OSCC cell-lines. S100A16 was over-expressed and knocked-down in OSCC-derived cells by retroviral vectors and concomitant modulation of differentiation markers was examined. **a** Western blot analysis showed up-regulation of several of the differentiation markers with S100A16 over-expression. ** anti human-cytokeratin 13 (sc-58721, Santa Cruz); *anti human-cytokeratin 13 (NCL-CK13, Novacastra). **b** Up-regulation of cytokeratin 13 in S100A16-CaLH3 was further verified by FACS analysis (**b**–**d**). Error bars in (**c** and **d**) represent SEM of 3 repeated experiments. Student’s-*t* test was performed for statistical analysis. **d** In parallel with over-expression, S100A16 knock-down led to down-regulation of involucrin and cytokeratin 13 in CaLH3 cells

The *in vivo* and *in vitro* association of S100A16 with a more differentiated phenotype led us to investigate whether S100A16 can induce expression of differentiation-related markers in OSCC-derived cells. Retroviral mediated over-expression of S100A16 resulted in up-regulation of involucrin, cytokeratin 13 and transglutaminase 1 in CaLH3 cells (expression of filaggrin could not be detected in both control and S100A16-CaLH3 cells) (Fig. 4a). In H357 cells, over-expression of S100A16 was associated with up-regulation of involucrin, cytokeratin 10 and filaggrin (expression of transglutaminase 1 and cytokeratin 13 could not be detected in both control and S100A16-H357 cells (Fig. 4a). FACS analysis further confirmed the up-regulation of cytokeratin 13 upon S100A16 over-expression (Fig. 4b–d). Confirming the above results, shRNA mediated knock-down of S100A16 resulted in down-regulation of involucrin and cytokeratin 13 in CaLH3 cells (Fig. 4e). The total p38 or phospho-p38 expression levels were not affected by S100A16 over-expression (Fig. 4a).

### S100A16 over-expression reduced cell proliferation, sphere formation ability and 3D-invasive potential of OSCC-derived cells *in vitro*

**Fig. 5:**
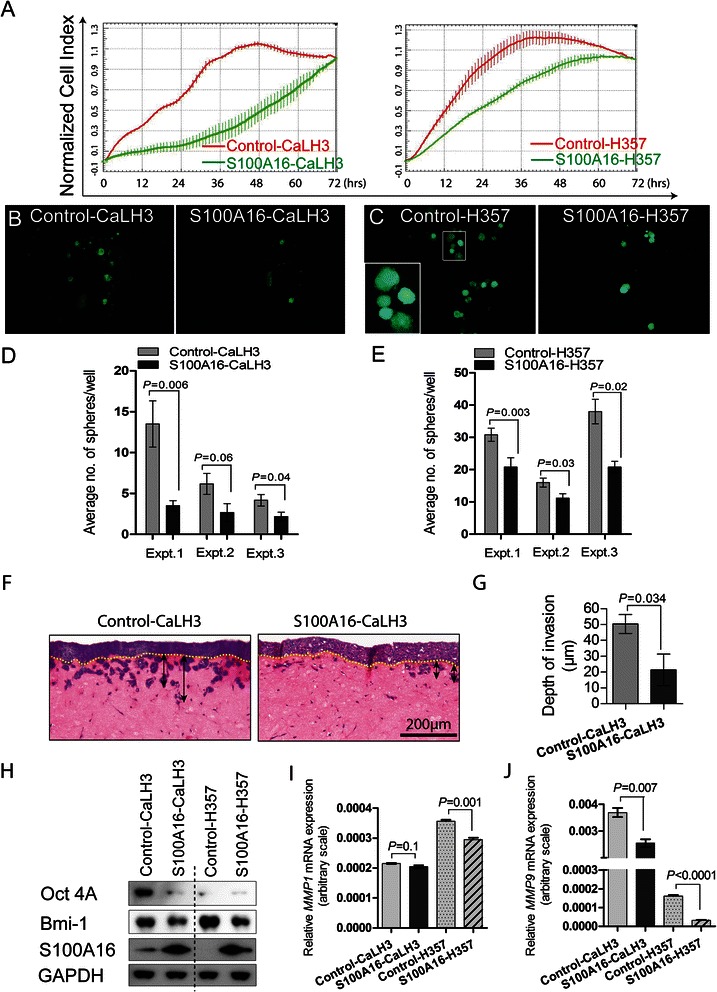
Retroviral mediated S100A16 over-expression inhibited proliferation, sphere formation and 3D-invasion abilities of OSCC cells *in vitro*. S100A16 was over-expressed in CaLH3 and H357 cell-lines and subsequent effect on malignant phenotype was examined. **a** Six thousands control or S100A16 over-expressing CaLH3 or H357 cells were seeded in duplicates in the microtiter E-plates and cell proliferation was measured in real time for 72 h with the xCELLigence system. S100A16 over-expressing cells proliferated significantly slower as compared to the control cells as demonstrated by the normalized cell index. Similarly, sphere formation abilities of S100A16-CaLH3 (**b** and **d**) and S100A16-H357 (**c** and **e**) cells were significantly reduced as compared to the corresponding controls. Error bars represent SEM of 6 replicates for each experiment. Student’s-*t* test was performed for statistical analysis. Expt., Experiment. S100A16 over-expression led to significant reduction in the invasive potential of CaLH3 cells in 3D-organotypic cultures (**f**, invasion quantified in **g**). Yellow dotted line represents the imaginary basement membrane. Black vertical lines with double arrowheads represent the depth of invasion of malignant keratinocytes. **h**–**j** S100A16 mediated functional effects on malignant phenotype were associated with concomitant down-regulation of proliferation/self-renewal markers (Bmi-1 and Oct4A) (**h**) and invasion-promoting molecules (*MMP1* and *MMP9* mRNA levels) in CaLH3 and H357 cell-lines (**i** and **j**). Error bars in (**g**) represent SEM of 4 repeated experiments where as in (**i** and **j**) represent 3 repeated experiments. Student’s-*t* test was performed for statistical analyses

The functional role of S100A16 in OSCC tumorigenesis was next examined by performing a number of established functional assays. Proliferation rates (as measured by normalized cell index) of CaLH3 and H357 cell-lines were found to be significantly reduced upon S100A16 over-expression (Fig. 5a). More importantly, over-expression of S100A16 led to significant reduction in the sphere formation abilities (*in vitro* surrogate for the *in vivo* tumorigenesis assay) of both CaLH3 and H357 cell-lines as compared to the corresponding control cells (Fig. 5b–e) (*P* < 0.05). Suppression in sphere formation abilities correlated with a simultaneous down-regulation of self-renewal markers (Oct 4A and Bmi-1) in S100A16-CaLH3 and S100A16-H357 cells (Fig. 5h). Furthermore, S100A16 over-expression led to significant reduction of the invasive potential of CaLH3 cells in 3D-organotypic cultures (Fig. 5f, quantified in g). In parallel, S100A16 over-expression led to significant down-regulation of *MMP9* mRNA levels in both CaLH3 and H357 cells-lines (Fig. 5j). *MMP1* mRNA expression, however, was significantly down-regulated only in H357 cells (Fig. 5i).

### S100A16 over-expression decreased tumor formation ability of H357 cells in NOD/SCID mice and the resulting tumor xenografts exhibited a more differentiated and less proliferative phenotype

**Fig. 6:**
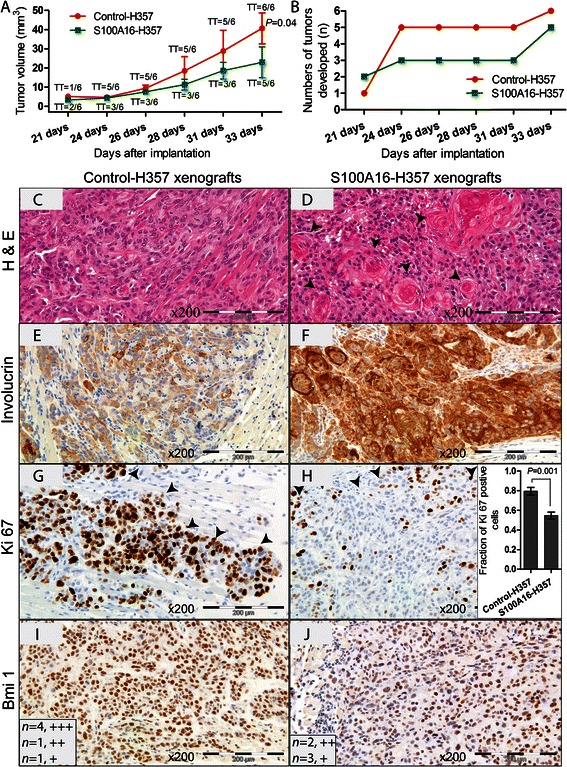
S100A16 over-expression reduced tumorigenesis *in vivo* and the resulting tumor xenografts exhibited features of increased differentiation. **a** One thousand control or S100A16 over-expressing H357 cells were injected in the tongue of each mouse (NOD/SCID) and tumor development was monitored. All mice injected with control cells formed tumors (Tumor Take, TT = 6/6) whereas S100A16-H357 cells formed tumors in 5 of the mice (TT = 5/6). In addition, tumors formed by control cells were significantly larger as compared to that of the S100A16-H357 cells (*P* = 0.04, at 33 days). Error bars represent SEM. Student’s-*t* test was performed for statistical analysis. **b** S100A16-H357 cells took longer time (lag phase) to form tumors as compared to the control cells. Tumor xenografts were harvested, formalin fixed, paraffin embedded and examined for histomorphology, IHC expression of differentiation, proliferation and self-renewal markers. Representative images demonstrating highly differentiated phenotype of S100A16-H357 xenografts with formation of keratin pearls (arrowheads) and higher expression of involucrin (**c–f**). On the other hand, S100A16-H357 xenografts demonstrated lower expression levels of Ki67 (**g** and **h**) and Bmi-1 (**i** and **j**) as compared to the control-H357 xenografts. Arrowheads in (**g**) and (**h**) mark the invading front. Error bars in (**h**) represent SEM. Student’s-*t* test was performed for statistical analysis in (**f**). +++, strong; ++, moderate; +, weak staining

The effect of S100A16 on the *in vivo* tumor formation ability was examined by injecting S100A16 over-expressing (S100A16-H357) or control (control-H357) H357 cells in the tongue of NOD/SCID mice. When 1000 cells/mouse were injected, control-H357 cells formed tongue tumors in all of the NOD/SCID mice (6/6, 100 % tumors) whereas S100A16-H357 cells formed tumors in 5 of the mice (5/6, 83.4 % tumors). More importantly, tongue tumors formed by the control-H357 cells were significantly larger (at 33 days, *P* = 0.04) compared to that of S100A16-H357 cells (Fig. 6a). In addition, lag phase for S100A16-H357 cells to form tongue tumors was longer than that of control-H357 cells (Fig. 6b). We next examined whether the phenotype of S100A16-H357 tumor xenografts would correlate with the expression of differentiation and proliferation/self-renewal markers. As expected, S100A16-H357 xenografts demonstrated features of well differentiation (presence of keratin pearls, Fig. 6c and d) with higher expression of the terminal differentiation marker involucrin as compared to the control-H357 xenografts (Fig. 6e and f) Additionally, S100A16-H357 xenografts expressed lower levels of Ki67 (Fig. 6g and h) and Bmi-1 (Fig. 6i and j) as compared to the control-H357 xenografts.

## Discussion

In the current study, assessment of whole tissue specimens of NHOM, ODL, OSCC and positive lymph node showed progressive down-regulation of both S100A16 protein and mRNA levels during OSCC progression (Figs. 1 and 2). These data were confirmed by analyzing *S100A16* mRNA levels in laser captured microdissected specimens and in three independent OSCC microarray datasets (Fig. 2). These findings indicate that the reduced level of S100A16 might be related to OSCC progression. Given the high probability of chromosomal rearrangement in 1q21 region (where *S100A16* is located) in human cancers, one of the mechanisms for S100A16 down-regulation in OSCC could be the deletion of *S100A16* locus in these lesions. Indeed, a recent work reported a loss in the *S100A16* locus in OSCC specimens from India and Sri Lanka [41]. The clinicopathological analysis of the current study showed a significant correlation between low S100A16 protein (at the invading front/island) levels and reduced 10-year overall survival probabilities for OSCC patients (Fig. 1e), poor tumor differentiation and positive cervical nodes. These data suggest a prognostic value for S100A16 in OSCC. Of note, S100A16 protein expression at the tumor center did not reveal any association with clinicopathological variables. These findings are in agreement with the concept that tumor invading fronts/islands are the more active areas of a malignant lesion and molecular/morphological changes at these areas are better prognosticators than those at the central/superficial region of the tumor [42, 43].

Positive correlation between S100A16 protein level and tumor differentiation, as found in the current study, pointed to a functional role of S100A16 in the regulation of keratinocyte differentiation. In agreement, mRNA levels of *S100A16* and differentiation markers were positively correlated in the OSCC specimens *in vivo* in two independent microarray datasets (Fig. 3a–d, Additional file 4: Figure S3) and in the cell fractions enriched for differentiated cells *in vitro* (Fig. 3e–g). Furthermore, modulation of earlier and terminal differentiation markers in OSCC-derived cells by over-expression and knock-down of S100A16 provided direct evidence that S100A16 functions as a differentiation promoting protein in OSCC (Fig. 4). This corroborates well with the observation that other members of S100 proteins are involved in the regulation of cellular differentiation [10, 11]. Indeed, a gradual increase in S100A16 expression has been described previously during the differentiation of preadipocytes to adipocytes [44]. However, in malignant oral keratinocytes this function seems to be independent of the p38 MAP kinase pathway, as no change was observed in the current study on the p38 phosphorylation status with S100A16 over-expression (Fig. 4a). This warrants investigation of p38 independent mechanisms possibly involved in S100A16 mediated modulation of differentiation markers in oral cancer cells.

Poorly differentiated phenotypes with excessive cellular proliferation and invasive abilities are considered to be characteristics of aggressive tumors. For several tumor types, lesions with more differentiated phenotype have been shown to have a less aggressive behavior and better clinical outcome [45, 46], indicating that molecular regulators that promote cellular differentiation might have tumor suppressive functions [47–49]. Several observations in the current study suggested that loss of S100A16 might contribute to the acquisition of aggressive OSCC phenotype. Firstly, significantly reduced S100A16 expression at the invading front/island as compared to the tumor center and, severely down-regulated expression in the positive lymph nodes indicated that loss of S100A16 might be necessary for the tumor cells to acquire an invasive phenotype (Figs. 1 and 2e). Further, correlation between reduced S100A16 expression level and reduced OSCC patient survival pointed towards a role for S100A16 in the maintenance of less aggressive tumor phenotype. Indeed, retroviral mediated S100A16 over-expression significantly suppressed several aspects of aggressive tumor phenotype; such as proliferation, sphere formation and 3D-organotypic invasive abilities of OSCC-derived cells *in vitro* (Fig. 5a–g). In parallel, S100A16 over-expression reduced tumorigenic abilities (tumor incidence and tumor volume) of H357 cells in *NOD/SCID* mice (Fig. 6a and b). These tumor suppressive functions were paralleled at the molecular level by concomitant down-regulation of self-renewal (Bmi-1and Oct4A) and invasion related (*MMP1* and *MMP9*) molecules *in vitro* (Fig. 5h–j). Likewise, S100A16-H357 xenografts were found to be more differentiated and less proliferative, both histologically and at the molecular level as evidenced by the higher involucrin expression and lower expression of Ki67 and Bmi-1 (Fig. 6c–j). Taken together, these findings suggested a role for S100A16 as a tumor suppressor in OSCC and indicated that progressive loss of S100A16 might be related with aggressive tumor growth and invasion leading to reduced patient survival. Similar to our results, IRF6 (INF regulatory factor 6), a pro-differentiating factor which shares similar expression pattern to that of S100A16, has been shown to have a tumor suppressive activity in squamous cell carcinoma by promoting keratinocyte differentiation [47, 49]. Additionally, progressive down-regulation of *S100A16* as found in CRC, prostate and ovarian cancers (Additional file 3: Figure S2) demonstrates a broader relevance for S100A16 in the process of tumorigenesis of other human malignancies and warrants further investigation in these tumor types.

## Conclusion

Our results indicate a novel role for S100A16 in the regulation of OSCC differentiation and tumor suppression. Further molecular characterization of S100A16 mediated tumor suppressive functions might contribute to the better understanding of OSCC carcinogenesis and provide opportunity for S100A16 based better prognostication and management of OSCC.

## Additional files

Additional file 1: **Supplementary Methods and Tables**. **Table S1**. S100A16 expression and clinicopathological variables of the OSCC patients. **Table S2**. Details of the TaqMan assays used for qRT-PCR. **Table S3**. Details of the antibodies used for immunoblotting. (DOCX 31 kb)
Additional file 2: Figure S1. (A) S100A16 staining was strong with membranous localization at the invading front/island of well-differentiated OSCC. (B) S100A16 staining was very weak or absent in the infiltrating tumor islands of positive cervical lymph nodes. (TIFF 2613 kb)
Additional file 3: Figure S2. Down-regulation of *S100A16* mRNA levels in tumor tissues as compared to the normal controls in independent microarray datasets. (A) ESCC, (B) CRC, (C) prostate cancer and (D) *in vitro* progression model of ovarian cancer (MOSE-E, non-tumorigenic; MOSE-I, intermediate and MOSE-L, aggressive malignant phenotype). For statistical analysis, paired-*t* test was performed in (A) and one way ANOVA test with Bonferroni Post-Hoc in (B–D). Error bars represent SEM. **, 0.001–0.01. ns, not significant. (TIFF 275 kb)
Additional file 4: Figure S3. Positive correlation between *S100A16* mRNA expression and differentiation markers in OSCC specimens. Expression data were obtained from external microarray dataset [20]. (TIFF 337 kb)

## Abbreviations

NHOM: Normal human oral mucosa
ODL: Oral dysplastic lesion
OSCC: Oral squamous cell carcinoma
IHC: Immunohistochemistry
qRT-PCR: Quantitative reverse transcription-polymerase chain reaction
ESCC: Esophageal squamous cell carcinoma
CRC: Colorectal cancer
