# Supplementary material for: S100A16 promotes differentiation and contributes to a less aggressive tumor phenotype in oral squamous cell carcinoma
Source: BMC Cancer. 2015 Sep 9;15:631. doi: 10.1186/s12885-015-1622-1 (PMC4564982; doi:10.1186/s12885-015-1622-1)
Supplement: Additional file 1: — Supplementary Methods and Tables. Table S1. S100A16 expression and clinicopathological variables of the OSCC patients. Table S2. Details of the TaqMan assays used for qRT-PCR. Table S3. Details of the antibodies used for immunoblotting. (DOCX 31 kb) [file 12885_2015_1622_MOESM1_ESM.docx]

**Supplementary Methods**

**IHC for mouse tumor xenografts**

Protocols for all animal studies were approved by the Norwegian Animal Research Authority (Project ID: 20124236). Tongue of each mouse were formalin fixed, paraffin embedded, cut into 5 micron sections and stained with HE, anti-S100A16 (11456-1-AP, Proteintech, 1:80 dilutions), anti- involucrin (NCL-INV, Novacastra, 1:500 dilutions), anti-Ki67 (M7240, clone MIB-1, DAKO, 1:1000 dilutions), anti-Bmi-1 (05-637, Millipore, 1:150 dilutions) antibodies. IHC for all of these antibodies were done essentially as described for human tissue specimens, except that citrate buffer pH 6 (S1699, DAKO) was used for the antigen retrieval of involucrin, Ki67 and Bmi-1.

**S100A16 IHC evaluation**

The P score (number of S100A16 positive cells) was determined as follows: 0, when there were 0 to 25% positive cells; 1, when there were 26 to 50 % positive cells; 2, when there were 51 to 75 % positive cells and 3 when there were >75 % positive cells. The L score (membranous or/and cytoplasmic localization of S100A16) was determined as follows: 2, if the proportion of cells with membrane to cytoplasmic staining was >1; 1, if the proportion was equal to 1; and 0.5, if the proportion was <0.5. The I score (intensity of S100A16 staining) was calculated as follows: 2, if the proportion of cells with strong to weak S100A16 intensity was >1; 1, if the proportion was equal to 1; and 0.5, if the proportion was <1. The final (PLI) score was calculated by multiplying the individual P, L and I scores and averaging PLI scores of the three evaluated fields. According to this scoring system, the NHOM is supposed to express the highest PLI score (12) with a maximum of P, L and I scores.

**Laser microdissection of FFPE specimens**

Briefly, 15 micron thick sections of FFPE specimens were placed on the glass slides (MembraneSlide NF 1.0 PEN, Zeiss, Germany) activated with UV light. Slides were then incubated at 56 °C for 2 hours, de-paraffinized in xylene, rehydrated in graded ethanol, stained with methylene green (S1962, DAKO), and dehydrated in reverse graded ethanol and xylene. Fifty to hundred µm^2^ tissue specimens of NHOM, ODL and paratumor epithelium, tumor center and the corresponding invading front/islands of each OSCC specimen were laser microdissected using a Zeiss Axiovert 200 inverted microscope equipped with a microlaser system (P.A.L.M Microlaser Technologies). Microdissected tissues were collected in nuclease free tubes (AdhesiveCap 500 clear, Zeiss) and subjected to RNA extraction.

**Construction of S100A16 expression and shRNA vectors and transfection**

Human cDNA encoding *S100A16* was amplified using primers pairs (F: 5' –ATCCCGCGGCAGGGAGATGTCAGACTGCTA-3' and R: 5'-TGAGGATCCCTAGCTGCTGCTCTGCTG-3') and subcloned into the pRetroX-IRES-ZsGreen1 retroviral expression vector (Clontech Laboratories, Inc., CA, USA). shRNA targeting *S100A1*6 mRNA was constructed using the following oligonucleotides: (F: 5’- GATCCCCGAACAAGATCAGCAAGAGCAGCTTCAAGAGAGCTGCTCTTGCTGATCTTGTTCTTTTTGGAAA -3’; R: 5’-AATTTTTCCAAAAAGAACAAGATCAGCAAGAGCAGCTCTCTTGAAGCTGCTCTTGCTGATCTTGTTCGGG -3’). Oligonucleotides were annealed and inserted in the RNAi-Ready pSIREN-RetroQ-DsRed-Express expression vector (cat. no: 632487, Clonetech). shRNA targeting *LacZ* gene was used as a control for the S100A16-shRNAs. Cancer cell-lines were infected with the retroviruses derived from packaging (Phoenix A) cells, sorted (DsRed as a marker), propagated, verified for knockdown of S100A16.

**RNA extraction, cDNA synthesis and qRT-PCR**

Frozen specimens of NHOM and OSCC were stored at -80 °C until mRNA extraction (Dynabeads mRNA Direct kit, Invitrogen) and cDNA synthesis (Transcriptor cDNA kit, Roche). RNeasy FFPE Kit (#73504, Qiagen) was used to extract RNA from laser microdissected tissues of NHOM, ODL and OSCCs. qRT-PCR amplification of *S100A16* mRNA was performed in duplicates in the LightCycler 480 qPCR system (Roche) using LightCycler^®^ 480 Probes Master (#04707494001, Roche).*GAPDH* and *ACTB* were used as endogenous controls.

Total RNA was extracted from the RAC, LAC and MAC cells and p75NTR^high^ and p75NTR^low^ CaLH3 cells using RNeasy fibrous tissue mini kit protocol (cat no: 74704, Qiagen Inc.). Following manufacturers’ instructions, 200-300 nanograms of total RNA was converted to cDNA using High-Capacity cDNA Archive Kit system (cat no: 4368814, Applied Biosystems). qRT-PCR amplifications was performed on ABI Prism Sequence Detector 7900 HT (Applied Biosystems) in triplicates as described previously [[1](#_ENREF_1)]. For details of the TaqMan assays used, see supplementary Table S1. *GAPDH* was used as endogenous control. Comparative 2^-ΔΔ Ct^ method was used to quantify the relative mRNA expression.

**Fluorescent activated cell sorting (FACS) analyses for P75NTR and cytokeratin 13**

For P75NTR cell sorting, cells were trypsinized, washed and resuspended in PBS containing 1% FBS and 1% HEPES buffer and incubated with mouse monoclonal anti-P75NTR antibody (Sigma Aldrich, 1:250 dilutions) for 10 minutes in ice. Mouse IgG1 (DAKO) was used as an isotype control. Alexa Fluor® 488 F(ab^1^)2 fragment of goat anti-mouse H+L (Invitrogen) was used as the secondary antibody. FACS sorting was done in BD FACSAria ^TM^ IIu (BD biosciences) using 550/50 BP Filter. The 4-5% of cells with the highest and the lowest expression of P75NTR were designated respectively as the p75NTR^high^ and p75NTR^low^ cell subsets. Post-sort was performed to ensure the quality of sorting.

For cytokeratin 13 staining, cells were trypsinized, washed and fixed with cold (-20 °C) methanol for 10 minutes, incubated with anti-cytokeratin 13 (Novacastra, 1:350 dilutions) antibody for 30 minutes at room temperature. Mouse IgG1 (DAKO) was used as an isotype control. Alexa Fluor® 647 goat anti-mouse H+L antibody (Invitrogen) was used as secondary antibody. FACS analysis of the stained cells was done in *Accuri6* cytometer (BD Biosciences). All FACS analyses were repeated three times and at least 10000 events were analyzed for each sample.

**Reference for supplementary methods:**

1. Sapkota D, Bruland O, Costea DE, Haugen H, Vasstrand EN, Ibrahim SO: **S100A14 regulates the invasive potential of oral squamous cell carcinoma derived cell-lines in vitro by modulating expression of matrix metalloproteinases, MMP1 and MMP9**. *Eur J Cancer* 2011, 47:600-610.

**Supplementary Tables**

Table S1. S100A16 expression and clinicopathological variables of the OSCC patiensts.

PLI score at tumor center*

Variables Low, n (%) High, n (%) *P*

Age** (years)

≤64 16 (53.3) 14 (46.7) 0.399

>64 15 (42.9) 20 (57.1)

Gender

Female 9 (42.9) 12 (57.1) 0.590

Male 22 (50.0) 22 (50.0)

Location

Tongue 13 (41.9) 18 (58.1) 0.669

Gingiva, buccal mucosa & oral lip 12 (52.2) 11 (47.8)

Floor of mouth & oro-pharynx 6 (54.5) 5(45.5)

Differentiation

Poor and moderate 17 (48.6) 18 (51.4) 0.878

Well 14 (46.7) 16 (53.3)

Lymph node involvement

Negative (N0) 18 (47.4) 20 (52.6) 0.951

Positive (N1 & N2) 13 (48.1) 14 (51.9)

Tumor size

T1 & T2 20 (55.6) 16 (44.4) 0.157

T3 & T4 11 (37.9) 18 (62.1)

Recurrence

No 20 (43.5) 26 (56.5) 0.290

Yes 11 (57.9) 8 (42.1)

Tumor stage

Early (1 & 2) 13 (61.9) 8 (38.1) 0.113

Late (3 & 4) 18 (40.9) 26 (59.1)

*OSCCs were stratified in to high and low S100A16 expression groups by using median S100A16 PLI score as a cut-off, ** patients were categorized into low- and high-age groups based on the median age.

Table S2. Details of the TaqMan assays used for qRT-PCR

Target Gene Protein encoded TaqMan assay ID

*S100A16* S100A16 Hs00293488_m1

*IVL* Involucrin Hs00902520_m1

*KRT10* Cytokeratin 10 Hs00166289_m1

*MMP1* MMP1 Hs00233958_m1

*MMP9* MMP9 Hs00957562_m1

*GAPDH* GAPDH Hs99999905_m1

*ACTB* Beta-actin Hs01060665_g1

Table S3. Details of the antibodies used for immunoblotting

Target Species Catalog / Soruce Dilution

S100A16 P (rabbit) 11456-1-AP / Proteintech 1/500

Involucrin M NCL-INV / Novacastra 1/200

Cytokeratin 13 M NCL-CK13 / Novacastra 1/50

Cytokeratin 13 M sc-58721 / Santa Cruz 1/200

Cytokeratin 10 M sc-53253 / Santa Cruz 1/200

Filaggrin P (rabbit) sc-30229 / Santa Cruz 1/200

Transglutaminase 1 P (rabbit) CVL-PAB0061 / Covalab 1/100

Bmi-1 M 05-637 / Millipore 1/1000

Oct 4 P (rabbit) sc-9081 / Santa Cruz 1/200

p38 P (rabbit) sc-7149 / Santa Cruz 1/200

p-p38 P (rabbit) sc-7149 / Santa Cruz 1/200

GAPDH P (rabbit) sc-25778 / Santa Cruz 1/5000

M monoclonal; P polyclonal
