# Supplementary figures and images for: S100A16 promotes differentiation and contributes to a less aggressive tumor phenotype in oral squamous cell carcinoma
Source: BMC Cancer. 2015 Sep 9;15:631. doi: 10.1186/s12885-015-1622-1 (PMC4564982; doi:10.1186/s12885-015-1622-1)

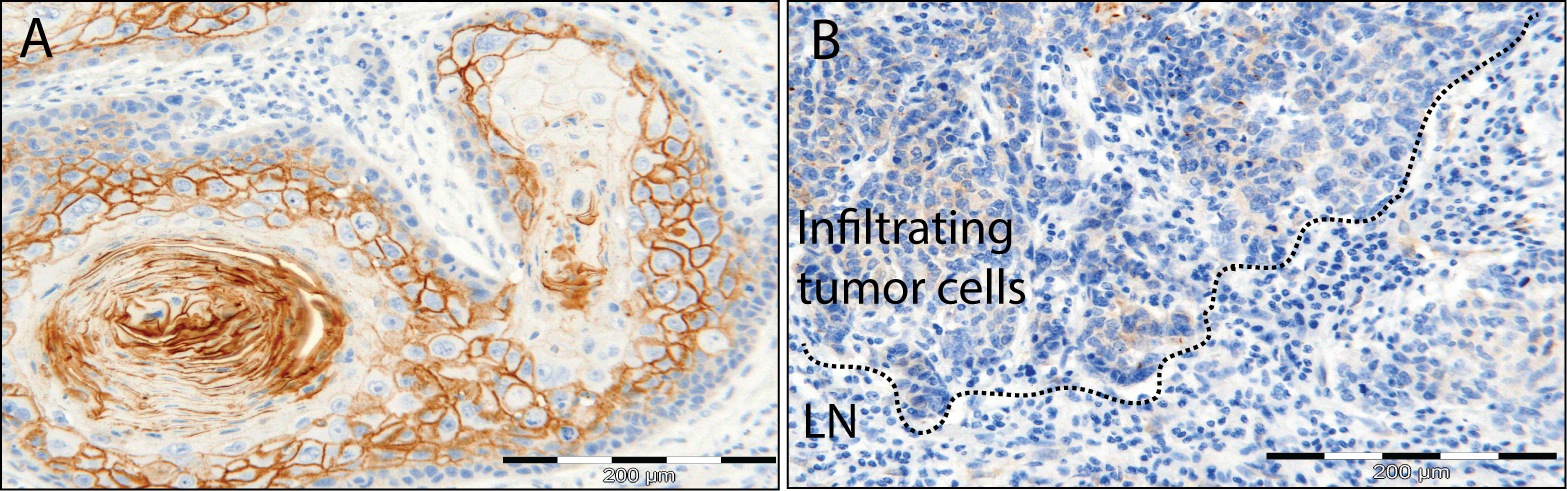

Supplement: Additional file 2: Figure S1. — (A) S100A16 staining was strong with membranous localization at the invading front/island of well-differentiated OSCC. (B) S100A16 staining was very weak or absent in the infiltrating tumor islands of positive cervical lymph nodes. (TIFF 2613 kb) [file 12885_2015_1622_MOESM2_ESM.tiff]
